# Laser Therapy for Infected Sites and Immediate Dental Implants in the Esthetic Zone: A Case Report and Review of Literature

**DOI:** 10.1155/2020/2328398

**Published:** 2020-01-07

**Authors:** Rolando Crippa, Riccardo Aiuto, Mario Dioguardi, María Peñarrocha-Diago, Miguel Peñarrocha-Diago, Francesca Angiero

**Affiliations:** ^1^Department of Oral Pathology, Italian Stomatological Institute, Milan, Italy; ^2^Department of Oral Surgery, University of Valencia, València, Spain; ^3^Department of Clinical and Experimental Medicine, University of Foggia, Italy; ^4^Department of Medical Sciences and Diagnostic Integrated, S. Martino Hospital, University of Genoa, Genova, Italy

## Abstract

Placement of postextraction dental implants has become a common practice. Here, we reviewed current literature, along with clinical procedures, outcomes, and incidence of complications, associated with immediate implants in infected postextraction sites. The YSGG (yttrium, scandium, gallium, and garnet) laser can significantly reduce the bacterial concentration after extracting a compromised tooth. We treated a 40-year-old woman with a compromised tooth in the esthetic zone, presenting clinical and radiological signs of infection, particularly a periapical periodontitis. The tooth was extracted after administering local anesthesia using Optocain® (mepivacaine and adrenalin 1 : 100,000), following which the site was treated with an ErCr : YSGG (erbium, chromium-doped yttrium, scandium, gallium, and garnet) 2780 nm laser device (Biolase iPlus®). The implant (Straumann® fixture) was inserted with minimum 35 N torque, 1 mm below the most apical bone peak. Bio-Oss® and resorbable membrane were applied to improve bone healing. The use of ErCr : YSGG laser has ensured success of implant therapy performed on an infected site. There were no complications such as peri-implantitis or loss of peri-implant bone. The implant achieved good primary stability, immediate placement into an infected site did not increase complications, and the 5-year follow-up confirmed the treatment success.

## 1. Introduction

Placement of postextraction dental implants has become a common practice, due to its numerous advantages, such as it facilitated maintenance of the horizontal and vertical dimensions of the osseous tissues [1], reduced treatment times, enhanced patient comfort, and good esthetic results. The immediate implant placement technique was first described by Lazzara in 1989 [2]. However, only a small number of studies report the clinical outcomes of immediate implants inserted in postextraction sockets.

One of the primary indications to this technique is the need to replace endodontically compromised teeth in cases when periapical surgery is inadvisable [3]. In such cases, it is imperative to note that certain local and systemic factors may contraindicate placement of the dental implant [4]. Recent studies have demonstrated that the presence of a periradicular infection may not compromise immediate implant placement, provided that the site is adequately decontaminated with a disinfection protocol [5]. The YSGG laser can significantly reduce the bacterial concentration present in the socket of an extracted tooth [6].

A number of studies have reported high success rates for immediately placed, in some cases immediately loaded, implants that are inserted in infected or inflamed postextraction sites [7]. However, to ensure the success of this technique, it is imperative to establish certain preoperative and postoperative measures, such as meticulous cleansing, alveolar debridement, administration of antibiotics, and postoperative 0.12% chlorhexidine mouth rinses [8].

Recent studies have reported that laser technology is capable of eliminating bacteria more effectively than chemical products. Kusek suggests that the hydroacoustic phenomenon, which combines bactericidal effects with the ability to reach anatomically complex regions, is the principal factor that ensures complete disinfection [6]. The article reviews the studies concerning the immediate implant technique after laser disinfection and presents a clinical case to illustrate the main steps for correct management of the procedure and the 5-year follow-up.

## 2. Case Report

**Figure 1:**
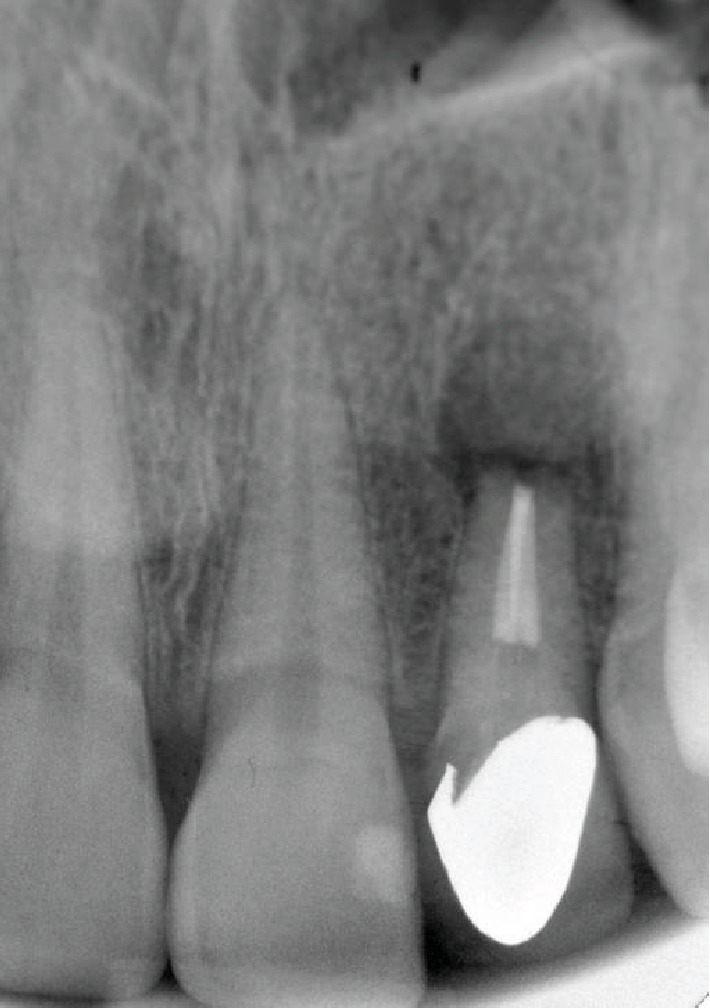
Preoperative X-ray.

**Figure 2:**
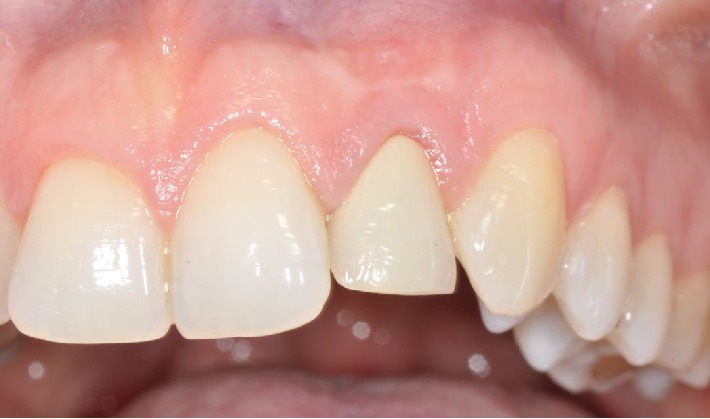
Preoperative clinical condition.

We encountered the case of a 40-year-old woman with a compromised upper left lateral incisor presenting with clinical and radiological signs of an infection, particularly periapical periodontitis (Figures 1 and 2). The tooth had been unsuccessfully treated with apicectomy. The patient was in good general health and had a good oral hygiene and was motivated to begin the treatment. We decided to proceed with a postextraction dental implant, considering the conditions and the area of high esthetic value.

**Figure 3:**
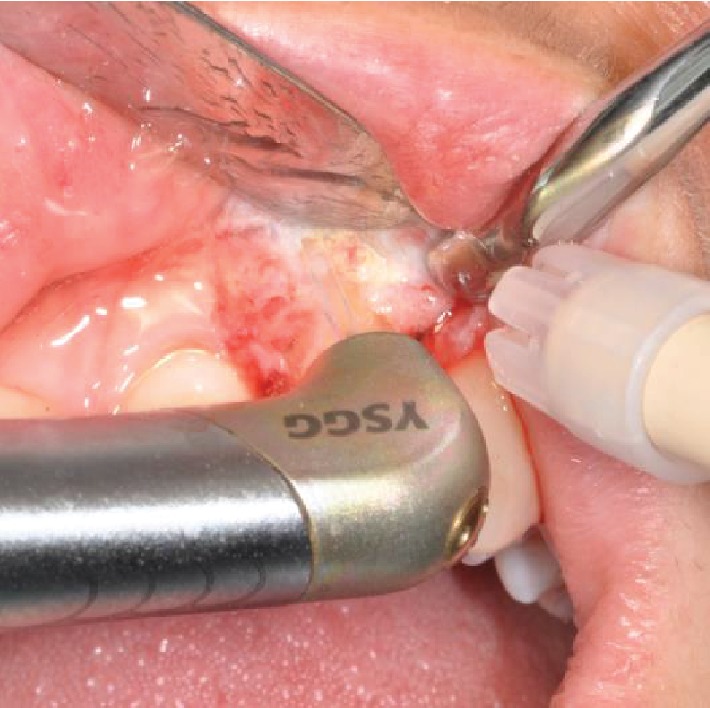
Phases of surgery.

**Figure 4:**
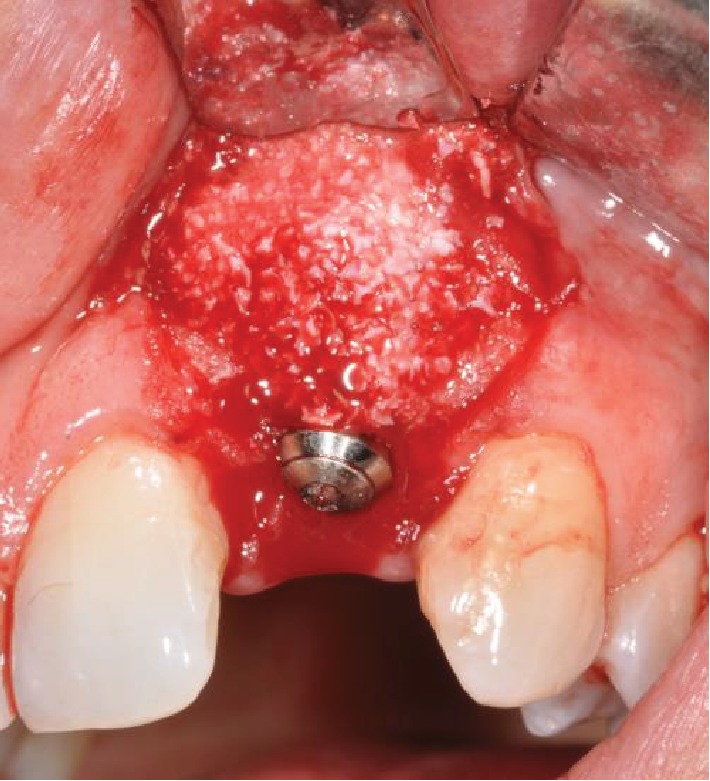
Tissue regeneration.

**Figure 5:**
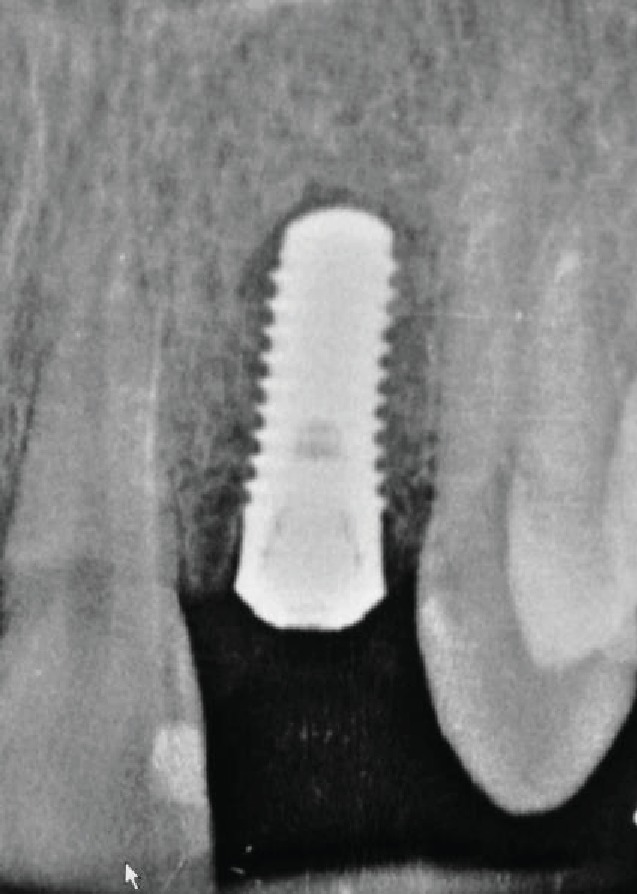
Postoperative X-ray.

**Figure 6:**
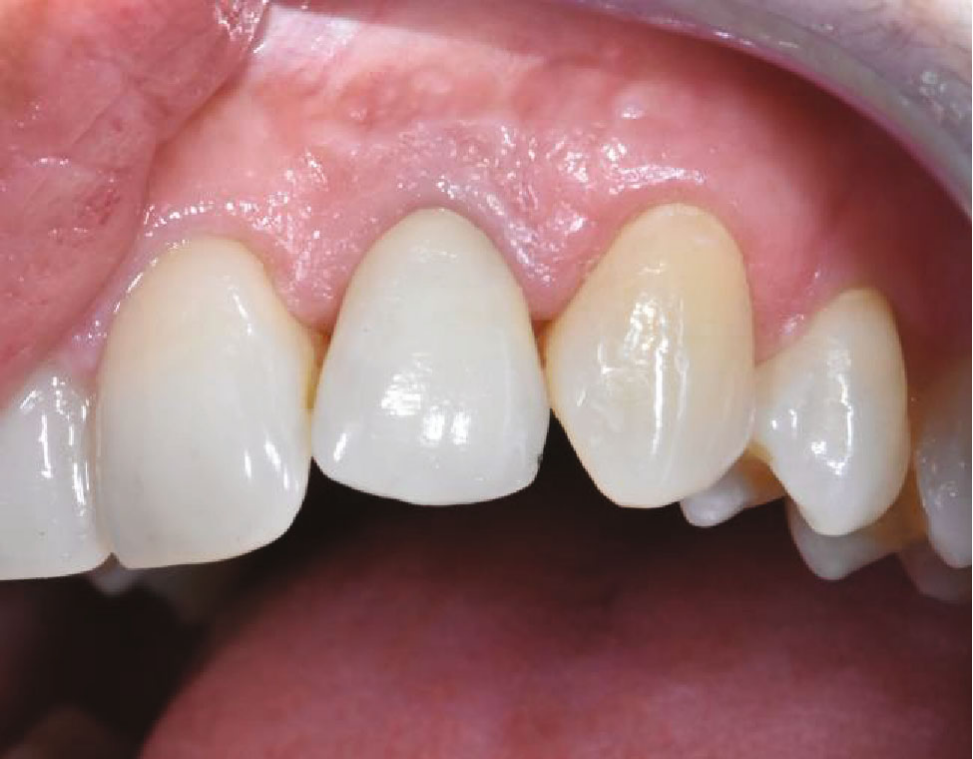
Clinical conditions after 2 years.

**Figure 7:**
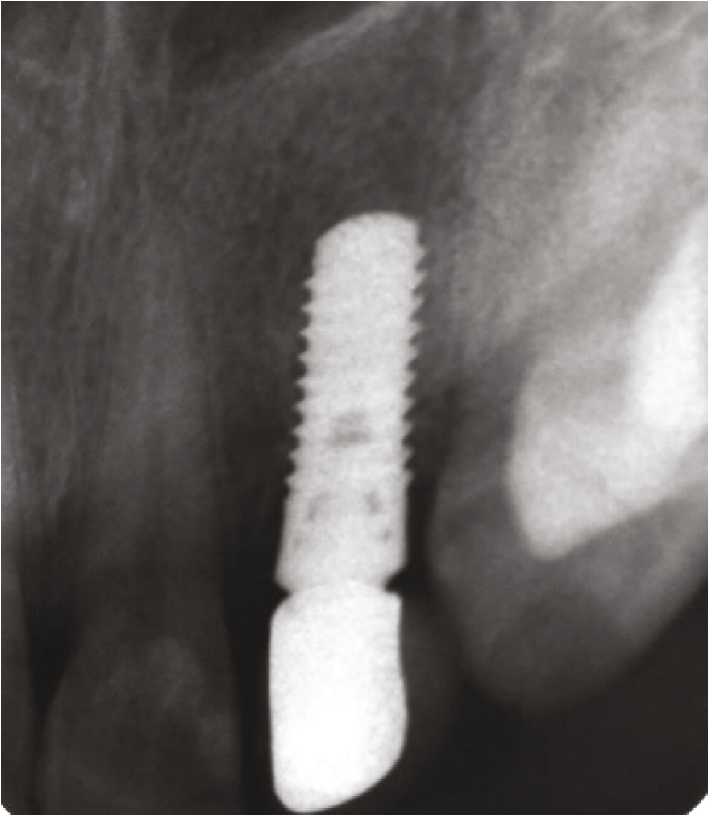
1-year follow-up.

Optocain® (mepivacaine 1 : 100.000) was used as local anesthetic, and tooth 2.2 was extracted as atraumatically as possible. The full thickness flap was carried out by a crestal incision with vertical releases. The postextraction site was treated with the ErCr : YSGG 2780 nm laser device Waterlase iPlus® (Biolase) with handpiece gold having two modes of operation, namely, the soft tissue and hard tissue modes (Figure 3). Configuration for the soft tissue mode includes tip MC-3, length 9 mm, air 20%, and water 40%; alternatively, the configuration for the hard tissue mode includes tip MZ-8, length 9 mm, air 40%, water 60%, 3.5 W, and 60 Hz. The site was debrided and decontaminated after extraction using the same laser device (2.0 W, 15 Hz, 40% air, 60% water, and 100 mL H_2_O/min in hard tissue mode) while mounting a MZ-6 tip and 9 mm in length. Debridement time depended on the amount of pathological tissue and bone volume, whereas decontamination lasted from 60 to 90 seconds per socket, ensuring no physical contact between the tip and the tissues. Straumann® fixtures were selected for the implant surgery. The implant (1 T.E. ø 3.3 mm RN, SLA®; 10 mm, Roxolid®) was inserted with a minimum 35 N torque and 1 mm below the most apical bone peak. Bio-Oss® and GUIDOR matrix barrier (DeOre Materials®) were used to improve bone healing (Figures 4 and 5). The suture was placed with particular care to obtain primary closure over the implant. The suturing material used was PTFE Omnia 3/0, 19 mm 3/8. We postoperatively administered amoxicillin (1 gr ×2/day for 6 days) and chlorhexidine gluconate 0.20% twice daily for 15-20 days. The temporary prosthetic phase before loading was managed with a Maryland bridge. The implant was loaded after 4 months, and a clinical check 2 years later demonstrates satisfactory esthetic outcomes (Figure 6). Radiographic checkups were scheduled on the 1^st^, 4^th^, 8^th^, and 12^th^ months in the first year (Figure 7).

## 3. Discussion

**Figure 8:**
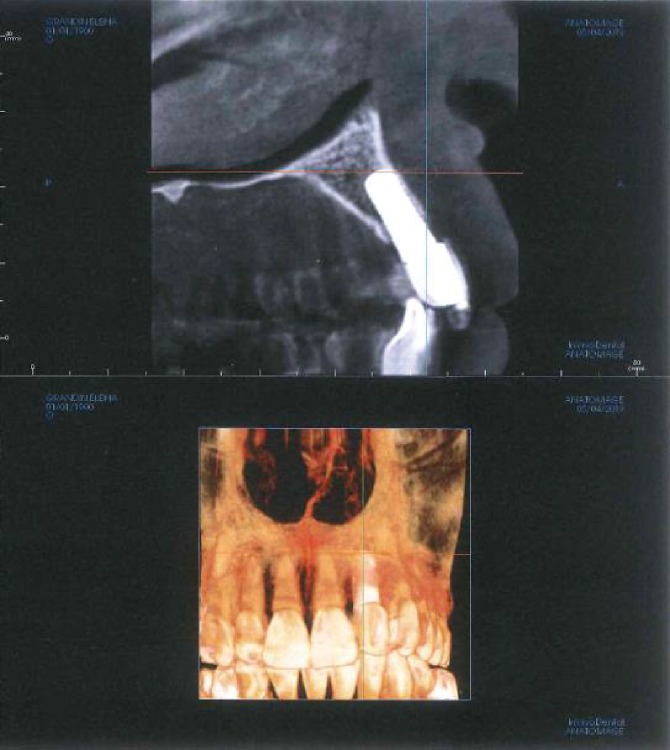
5-year follow-up.

We did not observe any complications, such as implant loss, peri-implantitis, or loss of the peri-implant bone. The implant achieved a good primary stability (>35 N/cm) and indicated that immediate placement into infected sites does not lead to more number complications than the traditional technique. This is evidenced by the 5-year follow-up (Figure 8) and research performed to analyze the scientific literature. The PICO assessment worksheet was used to define the topic and plan the search strategy, before commencing the review [9]. We searched for the studies including those limited to the period from January 1, 1980, to June 30, 2019. Furthermore, we used a specific set of keywords such as “immediate implant placement” AND “laser”, “dental implants” AND “laser” AND “postextraction”. The search was restricted to the study subjects due to the use of Boolean connectives. We used the PubMed (Medline) search engine and the NCBI database. All types of studies published in dental journals were considered.

Although the postextraction implant placement technique has been widely validated, little has been reported concerning the applications of laser decontamination of the infected sites for immediate implant placement. A search through the published studies produced only four clinical articles that combined laser treatment and immediate implant therapy (Table 1). Kusek presented 10 cases of immediate implant placement subjected to the ErCr : YSGG laser disinfection therapy and affirmed that these cases would have taken 3 times longer to heal if treated through traditional methods. Using this technique would therefore enable both the patient and dentist to benefit from a reduced treatment time [6].

Montoya-Salazar et al. also reported a similar study: they analyzed 36 immediate implants replacing teeth lost due to chronic periapical lesions, with a history of endodontic failure, and concluded that this therapy may be considered safe option to restore fresh infected postextraction sockets, provided that a strict debridement protocol was respected. Their protocol comprised curettage, cleansing with 90% hydrogen peroxide, irradiation with ErCr : YSGG laser, and chlorhexidine rinses, together with guided bone regeneration under antibiotic cover [7].

Crippa et al. described a series of 94 postextraction implants with a follow-up from 6 months to 4 years and a success rate of 94.6% (89/94) [10].

Additionally, Choi et al. described the advantages of using the laser for ridge conservation. However, that study was not pertinent to infected sites. The authors affirmed that using the Nd : YAG laser energy with 650 *μ*s pulse duration consistently supported rapid clot formation and graft containment at immediate implant and ridge preservation sites [11].

An important systematic review of the literature on immediate implants in infected sites was carried out by Waasdorp et al., but it does not include studies on the effects of laser decontamination [12].

Success after 5 years of follow-ups, the case described in this work reflects what the other authors observed in previous studies. According to the current scientific evidence, provided the presence of adequate primary stability, the implant immediate placement into infected sites would not present with increased rate of complications. However, according to the studies reviewed, it is imperative to conduct an RCT study on this objective, while following appropriate clinical protocol.

The effectiveness of the YSGG laser in disinfecting the surgical site depends on the photoacoustic effect of laser radiation, which attacks bacterial colonies [13, 14]. This effect has been extensively studied in vitro, through experiments that have demonstrated its validity [15, 16]. An important factor is the power setting of the laser: the power must be adjusted to ensure optimum disinfection of the site without risking collateral tissue damage [17]. Moreover, the operator's experience with this technique also plays a fundamental role. Using the laser device for implant surgery may also be advantageous in reducing intraoperative bleeding, therefore keeping the operative field clear [18].

## 4. Conclusion

Immediate implant placement in infected or inflamed postextraction sites, after laser decontamination, does not seem to increase the risk of failure, as demonstrated by this case and other previously published reports. The technique also offers interesting advantages of treating esthetic areas with postextraction implants. It is necessary to follow a certain set of protocols and procedures to prevent peri-implantitis and infective complications. However, further studies will undoubtedly be needed to fully elucidate the importance and mechanism underlying the technique.

## Figures and Tables

**Table 1:** Articles about laser treatment and immediate implant therapy.

Author	Study design	Infected sites	Laser	Implants (no.)	Follow-up	Survival rate
Kusek	Case series	Yes	ErCr : YSGG	10	1 year	10/10
Montoya-Salazar et al.	Prospective	Yes	ErCr : YSGG	18	3 years	17/18
Crippa et al.	Case series	Yes	ErCr : YSGG	94	6 months/4 years	89/94
Choi et al.	Case series	No	Nd : YAG	6	9 months	6/6
